# Quantitative analysis of waterfowl parvoviruses in geese and Muscovy ducks by real-time polymerase chain reaction: correlation between age, clinical symptoms and DNA copy number of waterfowl parvoviruses

**DOI:** 10.1186/1746-6148-8-29

**Published:** 2012-03-15

**Authors:** Grzegorz Woźniakowski, Elżbieta Samorek-Salamonowicz, Wojciech Kozdruń

**Affiliations:** 1 Department of Poultry Viral Diseases, National Veterinary Research Institute, Partyzantów 57 Avenue, 24-100 Puławy, Poland

## Abstract

**Background:**

Waterfowl parvoviruses cause serious loss in geese and ducks production. Goose parvovirus (GPV) is infectious for geese and ducks while Muscovy duck parvovirus (MDPV) infects Muscovy ducks only. So far, for these viruses' sensitive detection polymerase chain reaction (PCR) and loop-mediated isothermal amplification (LAMP) were applied. However, there was no molecular biology method for both waterfowl parvoviruses detection and quantification which could unify the laboratory procedures. The level of GPV and MDPV replication and distribution plays a significant role in the parvoviral infection progress and is strictly correlated to clinical symptoms. Meanwhile, experiments conducted previously on GPV distribution in geese, performed as animal trial, did not involve epidemiological data from the disease field cases. The study on the correlation between age, clinical symptoms and viral DNA copy number may be benefitable in understanding the GPV and MDPV infection. Such data may also aid in determination of the stage and severity of the infection with parvoviruses. Therefore the aim of this study was to develop quantitative real-time PCR for parallel detection of GPV and MDPV in geese and Muscovy ducks and to determine the correlation between the age of the infected birds, clinical symptoms and DNA copy number for the estimation of the disease stage or severity.

**Results:**

In order to develop quantitative real-time PCR the viral material was collected from 13 farms of geese and 3 farms of Muscovy ducks. The designed primers and *Taqman *probe for real-time PCR were complementary to GPV and MDPV inverted terminal repeats region. The pITR plasmid was constructed, purified and used to prepare dilutions for standard curve preparation and DNA quantification. The applied method detected both GPV and MDPV in all the examined samples extracted from the heart and liver of the infected birds. The conducted correlation tests have shown relationship between age, clinical symptoms during parvoviral infection and the DNA copy number of these pathogens. The method allowed for a sensitive detection of GPV and MDPV even in 1-week old infected goslings or 2-week old ducklings before observation of any disease symptoms.

**Conclusions:**

The developed method was found to be a valuable tool for the unification of laboratory procedures and both parvoviruses parallel detection and quantification. The conducted analysis revealed significant correlation between the age of the infected birds, the observed clinical symptoms and DNA copy number of GPV and MDPV in the examined organs. The obtained data may aid in better understanding of the pathogenesis and epidemiology of Derzsy's disease and 3-w disease as well as estimation of the infection's severity and stage of the disease.

## Background

Goose parvovirus (GPV) and Muscovy duck parvovirus (MDPV) cause substantial loss in the production of goslings and Muscovy ducklings. The infection with both parvoviruses spreads rapidly worldwide and causes high morbidity and mortality [1-3]. GPV and MDPV belong to *Dependovirus *genus of *Parvovirdae *family and cause Derzy's disease in geese or 3-w disease in Muscovy ducklings [3-6]. The emergence of the disease was observed in middle 60 s of 20^th ^century in European countries. The disease was noted among goslings and ducklings characterised by anorexia, wheezing, and locomotor dysfunction [1,2]. The specific lesions observed in the affected geese and ducks include myopathy of skeletal muscle, hepatitis, myocarditis, sciatic neuritis and polioencephalomyelitis [4]. Other commonly observed lesions include atrophy of lymphoid organs (bursa of Fabricius, spleen and thymus). The disease affects mainly young goslings and Muscovy ducklings between 2 and 4 weeks old. However, infections in older birds also occur and are often asymptomatic except ascites or loss in featheriness.

The capsids of GPV and MDPV are non-enveloped, 20-22 nm in diameter and assembled from 32 capsomers. Their genome is single-stranded DNA about 5106 nt (GPV) and 5132 nt (MDPV) [7-10]. The classical detection of GPV and MDPV by virus isolation in gosling or duckling embryos, cell cultures and serological assays like ELISA and seroneutralisation test (SN) is dependent on the availability of SPF gosling embryos and standard positive control sera [2]. These limitations caused an increase in application of polymerase chain reaction (PCR) based techniques which allowed for the identification of GPV or MDPV [11-13]. Recent reports by Ji et al. [14] and Jang et al. [15] presented application of loop-mediated isothermal amplification assay (LAMP) which remarkably simplified the detection of both viruses. In spite of the diagnostic power of these methods they remain qualitative and are incapable to quantitate DNA viral copy which is important from both pathogenic and epidemiological point of view. Quantification of GPV by real-time PCR in experimentally infected goslings was previously described by Bi et al. [16] and Yang et al. [15]. However, these reports did not mention detection and quantification of MDPV. Moreover, the described research was conducted as an experimental approach on goslings and did not include detection and quantification of GPV or MDPV from field cases of Derzsy's disease in goslings or 3-w disease in Muscovy ducklings. The main goal of the presented study was to develop the real-time PCR for parallel detection and quantification of GPV and MDPV. The advantage of a single quantitative real-time PCR is the unification of GPV and MDPV detection and quantification. The second goal was to apply the developed real-time PCR method in order to assume correlation between the age of the infected birds, the observed clinical symptoms and viral DNA copy number. The presented data may be beneficial for better understanding of the pathogenesis of both parvoviral infections and their relationship with the observed clinical symptoms or lesions.

## Results

### Analysis of age and observed clinical symptoms in goslings and ducklings

**Figure 1:**
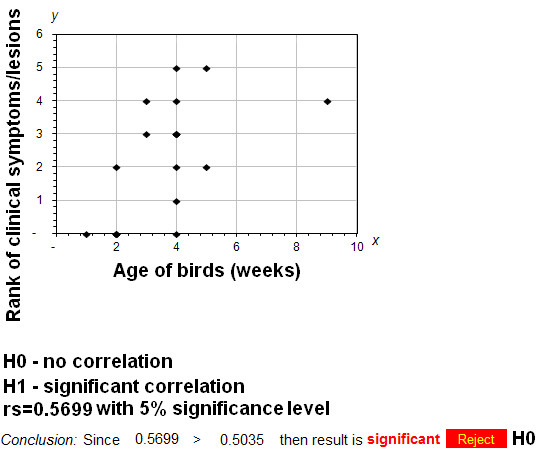
**Correlation between age of birds and observed clinical symptoms/lesions**. The rank values for clinical symptoms were presented on the y-axis while age of birds in weeks was plotted as the x-axis. Rs - Spearman's rank correlation coefficient, H0 - null hypothesis (no correlation between age of birds and clinical symptoms/lesions), H1 - alternative hypothesis (significant correlation between two examined data sets).

The conducted analysis of the age of goslings and ducklings and clinical symptoms specific for Derzsy's disease or 3-w disease has shown that symptoms manifested as a loss of featheriness and/or depression were mainly observed in birds older than 3 weeks (Figure 1). Similarly the most frequently observed ascites, pericarditis, kidney and liver enlargement or enteritis were found in birds older than 3 weeks (Table 1). The clear clinical symptoms like loss of featheriness, depression and ascites indicating 3-week disease were also detected in 9-weeks old ducks. However, no clinical symptoms or lesions were found in young goslings and ducklings aging up to 3 weeks. In the case of birds older than 5 weeks the clinical symptoms were less specific and manifested as a loss of featheriness and ascites. In spite of this fact the conducted Spearman's rank correlation test has shown a significant correlation between the age of the affected birds and the severity of the clinical symptoms and lesions since the calculated R_s _value reached 0.5699 which resulted in the rejection of the null hypothesis (Figure 1).

**Table 1:** The origin of GPV and MDPV isolates used in the conducted study and observed clinical symptoms and lesions in affected birds

Isolate	Age (weeks)	Species	Symptoms/lesions	Rank of symptoms/lesions
14/01	4	geese	Loss of featheriness, depression/ascites, enteritis, pericarditis	5
24/03	2	goslings	Not observed	0
33/03	1	goslings	Not observed	0
232/06	4	geese	Loss of featheriness, depression/ascites, pericarditis	4
8/07	3	goslings	Depression/ascites, enteritis, enlargement of kidneys	4
14/07	5	geese	Loss of featheriness, depression/ascites, enteritis, fibrinous pericarditis	5
16/07	4	geese	Depression/ascites, pericarditis	3
18/07	4	geese	Ascites, pericarditis	2
27/08	2	goslings	Enlargement of liver, pericarditiss	2
47/08	4	geese	Loss of featheriness/ascites, enlargement of kidneys	3
27/09	2	goslings	Not observed	0
30/09	3	goslings	Loss of featheriness, depression/ascites	3
52/09	9	ducks	Loss of featheriness/ascites, enlargement of kidneys	3
52/10	4	ducks	Depression/ascites, pericarditis	3
81/10	4	geese	Ascites, pericarditis	2
21/11	2	ducklings	Not observed	0

### Final conditions of real-time PCR for the quantification of DNA of GPV and MDPV

Final reaction conditions were optimized as follows: 12.5 μL 2× QuantiTect Probe PCR Master Mix (Qiagen, Germany), 0.5 μL of each primer (0.2 μM), 0.5 μL of *Taqman *probe (0.1 μM), 9 μL of deionised water and 2 μL of DNA template (~200 ng). The thermal programme was set as follows: 50°C/2 min., 95°C/15 min., 95°C/15 s, 60°C/60 s. The optimised real-time PCR assay was used for the detection and quantification of DNA copy number of GPV and MDPV.

### Standard plasmid pITR

Standard plasmid pITR was constructed for standard curve preparation and DNA copy quantification as well as sensitivity and specificity determination. Concentration of pITR plasmid was measured to be 1.2 μg/μL. The purity of the plasmid was determined at the wavelength of the absorbance of 260/280 nm to be 1.76. The calculated copy number of pITR stock plasmid was 3.5 × 10^11 ^copies/μL.

### Standard curve

**Figure 2:**
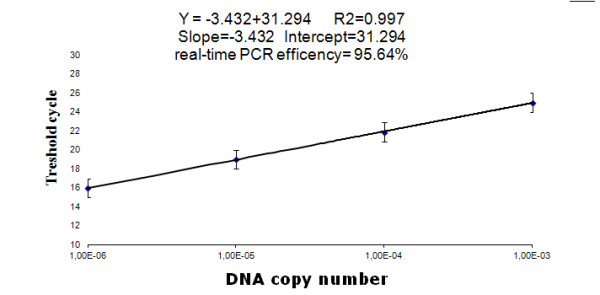
**Standard curve for the quantification of viral copy number by real-time PCR**. The used ten-fold dilutions ranged from 1.0 × 10^3 ^to 1.0 × 10^6 ^copies per μL and were plotted as X-axis. C_T _values are indicated on Y-axis. Standard deviation for each dilution of pITR plasmid was marked as "whiskers". The standard curve parameters calculation were given above the curve.

The obtained fluorescent curves and C_T _values of pITR serial 10-fold dilutions (from 10^3 ^up to 10^6^) copies were used for the construction of the standard curve for viral copy calculation in the examined DNA samples. The mean C_T _values for each dilution were: 24.988 for 1.0 × 10^3^, 21.86 for 1.0 × 10^4^, 18.97 for 1 × 10^5 ^and 15.94 for 1.0 × 10^6 ^pITR copies. The standard curve parameters calculation has shown that correlation coefficient value R^2 ^was 0.997 with slope = -3.432, intercept = 31.294 and efficiency E = 95.64%. By using standard formula for the regression analysis calculation Y = -3.423X + 31.294 it was possible to quantify the viral copy number in the examined samples (Figure 2).

### Sensitivity and specificity

**Figure 3:**
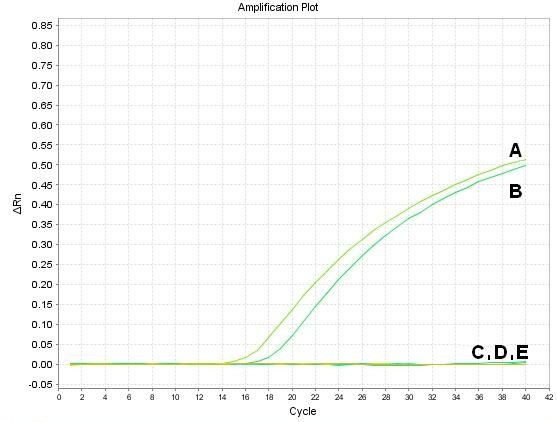
**Determination of specificity of quantitative real-time PCR**. A - DNA of GPV 88*v *strain, B - DNA of MDPV FM strain, C - DNA of goose circovirus (GCV), D - DNA of Avian adenovirus-1 (FAdV-1), E - DNA of goose haemorrhagic polyomavirus (GHPV). ΔRn is an increment of fluorescent signal during successive cycles.

The sensitivity determined on the basis of six 10-fold dilutions of pITR standard plasmid was tested in the reaction's final conditions. The detection limit was 1.6 × 10^1 ^copies of plasmid per reaction. The analysis of the DNA extracted from other waterfowl viruses has shown the developed method was specific only for GPV and MDPV isolates. The specificity plot was presented at Figure 3. No fluorescence signal was detected in negative control samples of GCV, GHPV and FAdV-1 which indicated the specificity of the assay. The constructed degenerated *Taqman *probe WfparvPro annealed to the complementary GPV and MDPV sequence of the ITR fragment.

### Correlation between age, clinical symptoms of the affected birds and GPV or MDPV copy number

**Figure 4:**
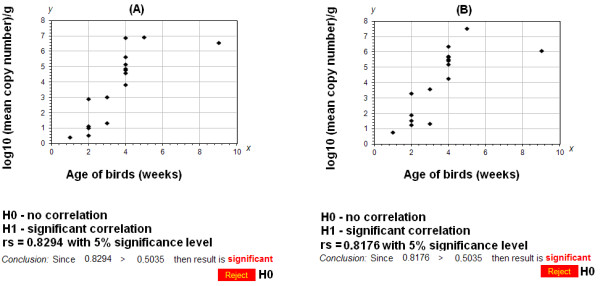
**Correlation between age of birds and DNA copy number in liver (A) and heart (B) of examined birds**. The copy number values were expressed as log_10 _per 1 g and were plotted as the y-axis while age of birds in weeks was presented on the x-axis. R_s _- rank Spearman's correlation coefficient. H0 - null hypothesis (no correlation between age of birds and DNA copy number), H1 - alternative hypothesis (significant correlation between two examined data sets).

The constructed standard curve allowed for the determination of copy number of GPV and MDPV in all the examined 16 field isolates (total 32 samples) from the heart and liver of the infected goslings and ducklings. The GPV copy number in goslings' liver ranged from 10^0.45 ^to 10^6.88^, and in goslings heart from 10^0.78 ^to 10^7.55 ^while in Muscovy ducklings the DNA copy number in liver ranged from 10^0.56 ^to 10^6.59 ^and 10^1.26 ^to 10^6.95 ^in heart (Table 2). The highest DNA copy number was detected in goslings and ducklings aging from 4 up to 9-w. These data were consistent with the observed clinical symptoms and lesions since in the case of birds older than 4-w the most frequently observed symptoms and lesions included: ascites, loss of featheriness, pericarditis, enteritis, liver enlargement and in three cases kidneys enlargement (Table 1). However, there was a minor difference in copy number in the case of birds older than 4-5-w. The DNA copy level in liver and heart of 1-2 w-old goslings was low and did not exceed 10^3 ^copies. This was also confirmed by the lack of any clinical symptoms in young goslings. Concluding this part of the study the performed correlation analysis was consistent showing close relationships between the observed severity of the disease and DNA copy number of both parvoviruses in liver and heart of the affected birds (Figure 4, Table 3). The conducted Spearman's rank correlation test has shown a significant correlation between the age of the diseased birds and the copy number in both liver and heart. The calculated R_s _values was 0.8294 for the analysis of DNA quantities in liver and 0.8176 for heart which was above the critical level with 5% significance (0.5035) (Figure 4). The quantified DNA copy number of both parvoviruses was also consistent with the age and severity of the observed clinical symptoms or lesions.

**Table 2:** Correlation between age of GPV and MDPV infected birds and the number of viral DNA copy.

Isolate	Age of birds	Species	Mean copy number log_10_/g in liver	Standard deviation (SD)	Mean copy number log_10_/g in heart	Standard deviation (SD)
21/11	2	ducklings	2.93	± 0.13	3.32	± 0.42
33/03	1	goslings	0.45	± 0.21	0.78	± 0.17
47/08	4	geese	4.91	± 0.34	5.21	± 0.32
52/10	4	ducks	4.62	± 0.29	5.72	± 0.31
24/03	2	goslings	1.16	± 0.11	1.90	± 0.09
81/10	4	geese	5.66	± 0.40	5.70	± 0.43
30/09	3	goslings	1.34	± 0.11	1.34	± 0.13
14/07	5	geese	6.95	± 0.43	7.55	± 0.44
8/07	3	goslings	3.05	± 0.25	3.61	± 0.30
48/10	2	ducklings	0.56	± 0.08	1.26	± 0.09
14/01	4	geese	3.86	± 0.22	4.27	± 0.28
18/07	4	geese	4.80	± 0.34	5.47	± 0.32
27/08	2	goslings	1.04	± 0.13	1.56	± 0.13
52/09	9	ducks	6.59	± 0.36	6.08	± 0.25
16/07	4	geese	5.16	± 0.20	5.54	± 0.17
232/06	4	geese	6.88	± 0.32	6.38	± 0.28

The age of birds was given in weeks

**Table 3:** Sequences of primers and probe used for the detection of GPV and MDPV

Primer/Probe	Sequence 5'-3'	Position in GPV genome	Position MDPV genome
WfParvF	ACCGGAAGTCACGTGAC	256-273	34-51
WfParvR	GTTCGTTCGTTCGAACC	379-396	160-177
WfParvPro	6FAM-ACCGGAAGCA**Y**GTGACC GGAA-TAMRA	327-348	93-114

## Discussion

In spite of the prophylaxis against Derzsy's disease of geese and 3-w disease in Muscovy ducks infctions still pose a threat for the production of waterfowl. The comparison of both aetiological agents (GPV and MDPV) by southern hybridization has shown close relation between them in the nucleotide and aminoacid sequence of their genomes [8,10,17]. However, both parvoviruses have different host and antigenic features [1,10]. An important tool to control both diseases is their fast and firm diagnosis. The classical virus isolation methods require at least 2 weeks and are dependent on the laying season of goslings and ducklings [2,3]. Serological assays provide important data regarding anti-GPV or MDPV antibody level in goslings and Muscovy ducklings but they are also dependent on SPF goose embryos and prone to the influence of maternal antibodies in reproduction flocks [2]. The polymerase chain reaction (PCR) was the first molecular method used for waterfowl parvoviruses detection [12,13]. Another fast LAMP molecular method for the detection of MDPV was introduced by Ji et al. [14] and Jang et al. [15]. The described LAMP-based methods are the milestone in GPV and MDPV diagnosis. On the other hand PCR and LAMP methods do not allow for the quantification of GPV and MDPV. So far, there was no molecular method developed for parallel detection and quantification of both waterfowl parvoviruses. Real-time PCR is an alternative for the classical PCR and is comparable in speed and labour with the LAMP technique. The method also provides quantitative data which may be used in the in-depth analysis of the pathogenesis or epidemiological study what is non-achievable by PCR or LAMP. It was previously confirmed that real-time PCR is reliable, simple and reproducible [16,18,19]. In the presented study the developed real-time PCR allowed for the quantification of GPV and MDPV copy number therefore it can be used for the monitoring of the viral load and determination of the possible stage of both diseases and their correlation with the age and the observed clinical symptoms or lesions in birds. Baigent et al. [20] used the quantitative real-time PCR in order to find the correlation of the CVI988 attenuated strain of Marek's disease virus load and its distribution in visceral organs of chickens. The presented study has shown significant correlation with the infected birds age and the observed clinical symptoms or lesions. In young geese and Muscovy ducks aging up to 2-w no clinical symptoms were observed and also the level of DNA copy number was the lowest and ranged from 0.45 to 3.32 log_10 _copies/g. The specific symptoms and lesions of the disease observed as ascites, loss of featheriness, pericarditis, enteritis and liver enlargement were observed in birds aging from 3 to 5-w what was confirmed with quantitative real-time PCR data since the number of DNA copy in these birds reached from 1.34 up to 7.55 log_10_/g. This indicated the most intensive replication of GPV and MDPV in the liver and heart of these birds. A slightly lower copy number - about 6.59 log_10 _copies/g was also detected in 9-w old ducks suggesting a decrease in the rate of viral replication. On the other hand the observed low DNA copy number in young 1-2-w old goslings and ducklings may indicate early infection with GPV or MDPV and transovarial transmission. Meanwhile, the previously described reports conducted on the experimentally infected goslings revealed the peak in GPV replication between 48 and 72 h post inoculation (PI) [19]. Bi et al. [16] found that fluorescent real-time PCR may be used for the detection and quantification of GPV in goslings as soon as 48 h PI. Similarly Limn et al. [11] detected GPV in liver, heart, brain, heart, pacreas and rectum of Muscovy ducklings as early as 2-d PI. The conducted studies on the infected goslings and ducklings from field farms have shown the field isolate of the virus was detected in 1-w old goslings while the highest viral load was observed in 4-5-w old birds. Yang et al. [19] and Limn et al. [11] described liver, spleen, thymus, Harder's glands and BF to be a good reservoir of GPV and MDPV. In the presented study due to standard laboratory diagnosis procedure the liver and heart of the examined birds were used. The DNA copy number in the examined liver and heart samples were comparable with the results obtained by Yang et al. [19]. Interestingly in the presented study the DNA copy number in young goslings and ducklings aging from 3-5 weeks were consistent with the observed clinical symptoms and pathological changes. Also the results described by Glávitis et al. (4) supported the data obtained in our study. The conducted analysis of correlation between the age of the examined birds and DNA copy number of GPV and MDPV gene revealed a significant lineage between them since the calculated R_S _values for liver and heart samples reached 0.8294 and 0.8176 which was above the critical level for the Spearman's rank correlation test (Figure 4). The obtained data are also consistent with the observed clinical symptoms and lesions in the affected birds. Moreover, these results were supported by the data obtained from the correlation test for the age of birds and the observed clinical symptoms or lesions (Figure 1).

## Conclusions

Concluding the conducted study the presented quantitative real-time PCR method may be applied as a routine procedure for veterinary laboratories for parallel detection and quantification of GPV and MDPV and may be beneficial for updating the actual data regarding pathogenesis, epidemiology and dynamics of GPV and MDPV replication during the infection in goslings and Muscovy ducklings.

## Methods

### Goslings and Muscovy ducklings

The infectious material for further study was taken from goslings and ducklings obtained by Department of Poultry Viral Diseases at NVRI during the post-mortem examination. All conducted autopsy examinations were done according to international guidelines and recommendations. All examinations conducted in the National Veterinary Research Institute were approved by National Bioethics Committee (Lublin, Poland). The liver and heart tissues were weighed and manually homogenised. The weight of each sample was 25 mg and was homogenised in PBS (phosphate saline buffer, Medlab, Lublin, Poland).

### Correlation between age of birds and clinical symptoms

Correlation between the age of the examined birds and the clinical symptoms and lesions severity was calculated using Spearman's rank (R_s_) correlation one-tail test with 5% with significance level. The choice of one-tailed test was due to the predicted nature of GPV and MDPV replication and incidence of clinical sympthoms or lesions. The R_s _value was calculated according to the following formula:

$$R_{S}=1-\frac{6\Sigma d^{2}}{n^{3}-n}$$

where: d^2 ^- square of differences between ranks and n - number of cases. Ranks for clinical symptoms and lesions were calculated as following: 0 - absence of clinical symptoms/lesions to 1-5 - one to five different clinical symptoms and lesions. The obtained R_S _value was compared with the critical p value for this level (0.5035). The R_s _value was higher than the critical level which resulted in the rejection of the null hypothesis (H_0_) about no significant correlation between the age and symptoms in the examined birds. This promoted the alternative hypothesis (H_1_) about the observed correlation. Calculations were made in Microsoft Excel ver. 2007 (Microsoft, RedMond, Washington, USA) and plotted as graphs.

### Field isolates

Total DNA was extracted from homogenates of liver and heart from goslings and Muscovy ducklings collected from 16 farms according to the procedure for tissue homogenates (Qiagen, Hilden, Germany). The numbers of the used field isolates of GPV stand for: 14/01, 24/03, 33/03, 232/06, 8/07, 14/07, 16/07, 18/07, 27/08, 47/08, 27/09, 30/09, 81/10. The isolates of MDPV stand for: 52/09, 52/10 and 21/11.

### Standard strains of viruses and DNA templates

GPV 88_v _was obtained from strain collection of the Department of Poultry Viral Diseases at the National Veterinary Research Institute (NVRI) in Puławy, Poland. The MDPV FM standard strain was provided by CEVA-Phylaxia Corporate, Budapest, Hungary. Goose circovirus (GCV), avian adenovirus 1 (FAdV-1), goose hemorrhagic polyomavirus (GHPV) were used as negative controls and were chosen from the strain collection of the Department of Poultry Viral Diseases (NVRI) in Puławy, Poland. The DNA templates were extracted from 200 μL of viral stock solutions using Qiagen Mini Kit (Qiagen, Hilden, Germany).

#### PCR primer and probes

**Figure 5:**
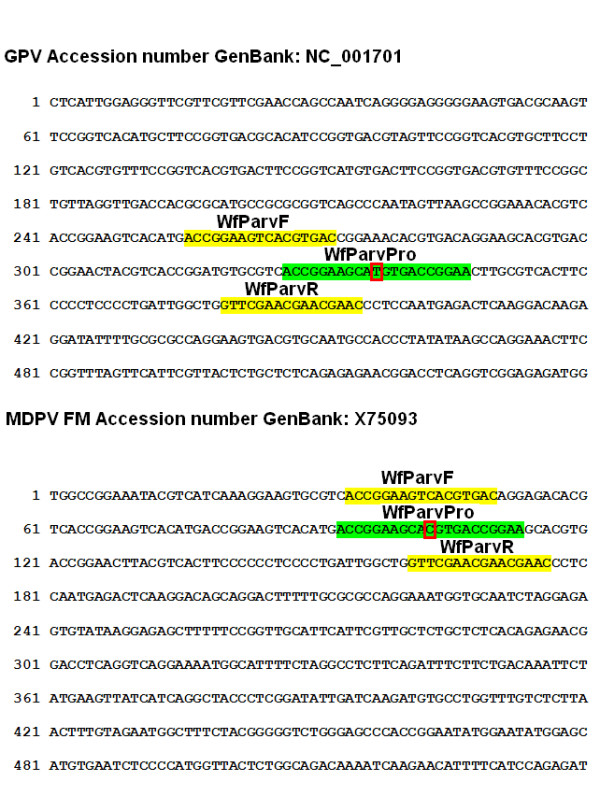
**Localisation of designed primers and probe for ITR region of GPV and MDPV**. The fragment of ITR was shown (from 1^st ^to 540^th ^nucleotide). Complementary sequences for primer were highlighted in yellow while for probe in green. The degenerated nucleotide position in *Taqman *probe sequence was marked with a red box.

Oligonucleotide primers and *Taqman *probe specific for ITR inverted terminal repeats region of GPV and MDPV were designed using Primer Express version 2.0.1 (Applied Biosystems, Foster City, California, USA) on the basis of complete sequence of the VG31/1 GPV strain (GenBank Accession No. EU583392) and the sequence of MDPV FM ITR region (GenBank Accession No. X75093). Due to single nucleotide differences in ITR region between GPV and MDPV the degenerated *Taqman *probe was designed with single Y nucleotide complementary to cytosine or thymine (C or T). The localisation of primers and probe with complementary regions was presented at Figure 5. The sequences of the primers and probe used were listed in Table 2. There were two product sizes: 123 bp for GPV and 126 bp for MDPV. The fluorogenic probe was labeled with the reporter: 6-carboxyfluoroscein (FAM) at the 5' end and with tetramethylcarboxyrhodamine (TAMRA) as a quencher at 3' end of the sequence. The probes and primers were synthesized by Genomed Co. (Warsaw, Poland) and purified by high-performance liquid chromatography [HPLC] system.

### Optimization of quantitative real-time PCR

The reaction was performed in 7500 Applied Biosystems thermocycler in 96-well tray (Applied Biosystems, Foster City, California, USA). The test was optimised using 4 primer concentrations (0.1, 0.2, 0.4 and 0.5 μmol/μL) and 4 probe concentrations (0.1, 0.2, 0.3, 0.5 μmol/μL). The reaction was conducted in 25 μL reaction volume in 96-well optical plates (Applied Biosystems, Foster City, California, USA). The data were collected by Applied Biosystems software ver. 2.0.1 (Applied Biosystems, Foster City, California, USA).

### Plasmid

The recombinant plasmid pITR was constructed by the PCR product insertion (123 bp) into pGEM-T Easy vector (Promega, Fitchburg, Wisconsin, USA). The primers used for the ITR fragment amplification were identical with the ones used for real-time PCR (Table 1). The construct was cloned into DH5α cells (Invitrogen, San Diego, USA) with blue/white screening according to procedures recommended by the producer. A single white colony harboring the plasmid with the ITR fragment was used for the inoculation of 5 ml of liquid LB medium with ampicillin (100 μg/mL), then the culture was incubated for 18 h at 37°C with shaking 225 rpm. The construct purity was determined by picking a single bacteria colony to PCR reaction mixture in the conditions described above. The plasmid DNA was extracted from 5 ml of inoculated media using Plasmid Mini Kit (Qiagen, Hilden, Germany).

### Construction of standard curve and regression analysis

Four ten-fold successive dilutions of the purified pITR plasmid with copy number ranging from 1.0 × 10^6 ^to 1.0 × 10^3 ^DNA/1 μL were used. The dilutions were prepared in PCR-grade purity water (Qiagen, Hilden, Germany). Each dilution was tested in triplicate and used for the construction of a standard curve by plotting a logarithm of copy number against cycle threshold values (C_T_). The standard deviation (SD) between replicates was also calculated and given on the standard curve plot. The values of regression coefficient R^2^, slope, intercept and reaction efficiency (E) were calculated by ABI 7500 system software ver. 2.0.1 (Applied Biosystems, Foster City, California, USA). The regression analysis was done using data exported to Microsoft Excel program ver. 2007 (Microsoft, RedMond, Washington, USA). For calculation of Y value used for the determination of viral copy number in the tested DNA samples the following equation was applied: Y = (slope)X + (intercept) where X - logarithm starting quantity and Y - threshold cycle. The viral load in the examined DNA samples extracted from the liver and heart of goslings and ducklings was calculated as a log_10 _from the mean of two replicates of each DNA sample per 1 gram of tissue.

### Real-time PCR sensitivity and specificity

The sensitivity of real-time PCR was tested in standard reaction conditions on the basis of six ten-fold dilutions from 1.0 × 10^6 ^to 1.0 × 10^1 ^copies of pITR plasmid. The detection limit of the assay was determined as the highest dilution which resulted in the presence of the fluorescent curve detected as C_T _value. The specificity was determined using DNA of 88_v _strain, MDPV FM, GoCV, FAdV-1 and GHPV from the strains collection of the Department of Poultry Viral Diseases at NVRI.

### Correlation between the age of birds and the copy number of GPV and MDPV

Correlation between the age of the examined birds and viral load in the examined DNA samples as a log_10 _per 1 gram of tissue was calculated using one-tailed Spearman's rank (R_s_) correlation test according to the manner described above for the clinical symptoms. The data were presented as separate graphs in Microsoft Excel ver. 2007 (Microsoft, RedMond, Washington, USA).

## Abbreviations

GPV: Goose parvovirus; MDPV: Muscovy duck parvovirus; 3-w disease: 3-week disease in Muscovy ducklings caused by MDPV; ELISA: Enzyme-linked immunosorbent assay; ITR: Inverted terminal repeated sequences flanking genomes of GPV and MDPV; pITR: pGEM-T plasmid containing fragment of ITR; nm: Nanometer; pg/μL: Pictogram per microliter; nt: nucleotides; SN: Seroneutralization assay; SPF: Specific pathogen free; R_s_: Rank Spearman's test; LAMP: Loop-mediated isothermal amplification; R^2^: Correlation coefficient for standard curve construction; GCV: Goose circovirus; GHPV: Goose hemorrhagic polyomavirus.

## Competing interests

The authors declare that they have no competing interests.

## Authors' contributions

GW carried out most of the described study and wrote the manuscript. WK provided standard strains and clinical observations used in the present study. ESS and WK participated in the study coordination. ESS and WK reviewed the final results and the manuscript. The final manuscript was read and approved by all authors.
